# Real-time robust generalized dynamic inversion based optimization control for coupled twin rotor MIMO system

**DOI:** 10.1038/s41598-022-21357-3

**Published:** 2022-10-25

**Authors:** Nadir Abbas, Xuejun Pan, Abdur Raheem, Rabia Shakoor, Zeeshan Ahmad Arfeen, Muhammad Rashid, Farhana Umer, Nouman Safdar, Xiaodong Liu

**Affiliations:** 1 School of Electrical Engineering, Dalian University of Technology, Dalian, 116024 China; 2 Department of Electrical Engineering, The Islamia University of Bahawalpur, Bahawalpur, 63100 Pakistan; 3 Department of Electrical Engineering, Government College University Faisalabad, Faisalabad, Pakistan

**Keywords:** Electrical and electronic engineering, Mechanical engineering

## Abstract

This work is used to design a novel robust optimization control law augmented with Robust Generalized Dynamic Inversion (RGDI) for continuous varying perturbations in the Twin Rotor MIMO System (TRMS). The perturbations like coupling effect, un-known states, gyroscopic disturbance torque, parametric uncertainties and parametric disturbances are considered as unwanted signal which should be optimized by an efficient controller. The variable structured systems like the TRMS (prototype) have great focus due to its high computational cost with a higher order non-linear behavior. The RGDI based controller designed to remove nonlinear dynamics as well as to avoid singularity issue with the augmentation of stability based mathematical operations (lyapunov stability analysis, controllability and observability matrices ) in the presence of considered perturbations during implementation. In this paper, we develop estimation of state deviation calculation between control angles and desired angles known as Euclidean error norm. The next step was to design RGDI based controller [Sliding Mode Control (SMC) and ${H_\infty }$ optimization] to minimize considered perturbations as well as the computational cost. The sharp (rapid) chattering phenomena in RGDI based SMC reduce the actuators performance that goes towards the failure of actuators. While the RGDI based ${H_\infty }$ optimization overcome the computational cost and minimizes ${H_\infty }$ norm that’s guaranteeing the robust stability as well as robust performance. The robustness of the optimization control technique validated by taking its worst case via MATLAB-Simulation. A real-time implementation applied to evaluate the worth of novel dynamic approach.

## Introduction

The control engineers always have focused the Variable Structure System (VSS) due to their highly nonlinear behavior, time-varying dynamics, coupling effect and sensitive towards perturbations during optimization control. The Unmanned Aerial Vehicle (UAV) is an extremely difficult task to control in the presence of all internal and external disturbances. Such systems are focused due to their extending applications in a narrow environment for civil security and military operations^1,2^. Twin Rotor MIMO System (TRMS) is a type of UAVs. Their ability to tilt their angle of flight, hovering, take-off and landing in irregular locations provide special interest to researchers^3,4^. A prototype of the TRMS resembles to a helicopter which can be served as an effective tool for experiments in a real-time environment^5^. The highly-coupled, a higher degree of nonlinear dynamics, uncertainties and gyroscopic torque needs to be tackled by efficient robust dynamic controller. The control researchers are attracted towards such problems i.e., TRMS, due to its ongoing expanding applications. The linear, nonlinear and intelligent control strategies are discussed to understand the behavior of TRMS as well as considered disturbances effect. Proportional integral derivative, Particle swarm optimization based Proportional integral derivative^6,7^, and the Linear Quadratic Regulator with output feedback control are linear strategies implemented in Refs.^8,9^. The backstepping control strategy also implemented to understand the behavior of the prototype in the presence of parametric uncertainties^10^. Sliding Mode Control^11^, Integral sliding mode control and second-order sliding mode control are implemented explained in Refs.^12,13^ with brief introduction. Integral sliding mode control combined with the linear quadratic regulator is discussed as a comparison technique to understand the worth of applied strategy^14^. The Model Predictive Control evaluation calculated in Ref.^15^, against a MIMO system. Further, a learning-based adaptive MPC has been developed by a researcher in Refs.^16,17^. The adaptive neural networks backstepping control in Ref.^18^, elaborated and the adaptive fuzzy backstepping control discussed in Ref.^19^. Adaptive type-2 fuzzy backstepping control for the fractional-order nonlinear system also studied in Ref.^20^, to understand the worth of upcoming hot research in control. The combined design of a nonlinear control and a classical control represented in^21,22^. The main reason of the cross-coupling between the pitch and yaw dynamics is the rapid change in rotors speed. Some un-known states of the MIMO system during mathematical modeling, make its structure more complex for the mathematician. The Nonlinear Dynamic Inversion (NDI) is a feedback linearization tool for the TRMS, used to reduce the complexity of the mathematical model^23^. The nonlinearities are cancelled at any stability point by feedback linearization. The draw-back of this method is that there may ignore some important nonlinearities, singularity, square matrix inversion. The large systems always required an efficient modelling as well as numerical singularity avoidance. These limitations are tackled by Generalized Dynamic Inversion (GDI) and inverse problem solved by the non-square inversion^24^. The left inversion type method is used to define the linear differential equations and inverted by the method Moore–Penrose Generalized Inverse (MPGI) based on Greville Method^25^. The major task behind this mechanism is to overcome inversion issues as well as avoiding from blind cancellation of important non-linear terms. The RGDI based controllers are implemented in different aerospace applications and robotics as well^26^. The stability analysis based on controllability and observability matrix provided to ensure effective controller stability. The Lyapunov function provides a platform to verify the asymptotic stability of the nonlinear system. The designed controller strategy based on the Ordinary Differential Equations (ODEs)^27^. The weighted function (tuning parameter) is used to reject the disturbance at output and track the desired response, and gives the robust stability to the model additive perturbation. For the multiplicative model uncertainty, smooth response (robust response) obtained by the function *S* /*T*. The Contribution of the paper is,

- We proposed here a new dynamic efficient control strategy implementation for the coupled system to get optimized results as compared to present control schemes.
- In this paper, we develop the estimation of state deviation calculation between control angles and desired angles known as Euclidean error norm.
- The proposed strategy, provide efficient robust response via MATLAB/simulink in the presence of considered varying perturbations.
- Verify that the RGDI based sliding mode control strategy can never be suitable to get efficient response due to chattering phenomena, which cause a serious problems for actuators.
- Real-time implementation under worse conditions (noise and parametric variation provided to both rotors simultaneously with disturbance torque) validate the worth of novelty of the optimization.
- Some important suggestions for control engineers are provided on the basis of experimental validation, to understand nature of control design as well as system behavior.

The remaining of this paper have following sections as the mathematical modelling in “Twin Rotor MIMO System (TRMS)”, while NDI and RGDI control design for the singularity avoidance based on stability analysis provided in “Nonlinear dynamic inversion” and “RGDI control design for singularity issue”. The sliding mode control strategy applied with their simulation results provided in “Design of SMC and simulations” and “RGDI based H∞ optimization and simulations”, elaborate the optimization. The experimental set-up in “Experimental setup and system connections” and conclusion based on validated results presented in “Conclusion”.

**Table 1:** Nomenclature.

Expression	Description	Expression	Description
$\theta$	Pitch (elevation) angle	$\phi$	Yaw (azimuth) angle
$\tau _1$	Momentum of main rotor	$\tau _2$	Momentum of tail rotor
$I_1$	Main rotor inertia	$I_2$	Tail rotor inertia
$x \in R$	(real number) of states	$u \in R$	input signal
$\rho$	Euclidean error norm	*D*	Diagonal matrix
*Y*	Control vector	*P*	Projection matrix
$a_1$	Constant	$b_1$	Constant
$a_2$	Constant	$b_2$	Constant
$M_g$	Gravitational momentum	${B_{1\theta }}$	Frictional parameter
${B_{2\theta }}$	Frictional parameter	${B_{1\varphi }}$	Frictional parameter
${B_{2\varphi }}$	Frictional parameter	$k_{gy}$	Gyroscopic parameter
$k_1$	Gain of main motor	$k_2$	Gain of tail motor
$T_{11}$	Denominator constant of main motor	$T_{10}$	Denominator constant of tail motor
$T_{21}$	Denominator constant of main motor	$T_{20}$	Denominator constant of tail motor
$u_{h}$	Horizontal axis control input	$u_{v}$	Vertical axis control input
$I_v$	Inertial momentum of main rotor	$I_h$	Inertial momentum of tail rotor
$e_z (t)$	Tracking of pitch and yaw angles	2*DOF*	Two degree of freedom
$k_{H_h }, k_{H_v}$	Velocity gains	$k_{f_h }, k_{f_v }$	Frictional momentum
$R_V$	Returned torque of rotors	$G_d$	Disturbance of plant
$G_u$	Transfer matrix of control signal	$K_y$	Feedback matrix functions
$K_r$	Transfer function matrix of pre-filter	*r*, *d*	Reference input, input disturbance
$\Delta _F$	Fictitious perturbation	$S_o$	Output sensitivity matrix

## Twin Rotor MIMO System (TRMS)

**Figure 1:**
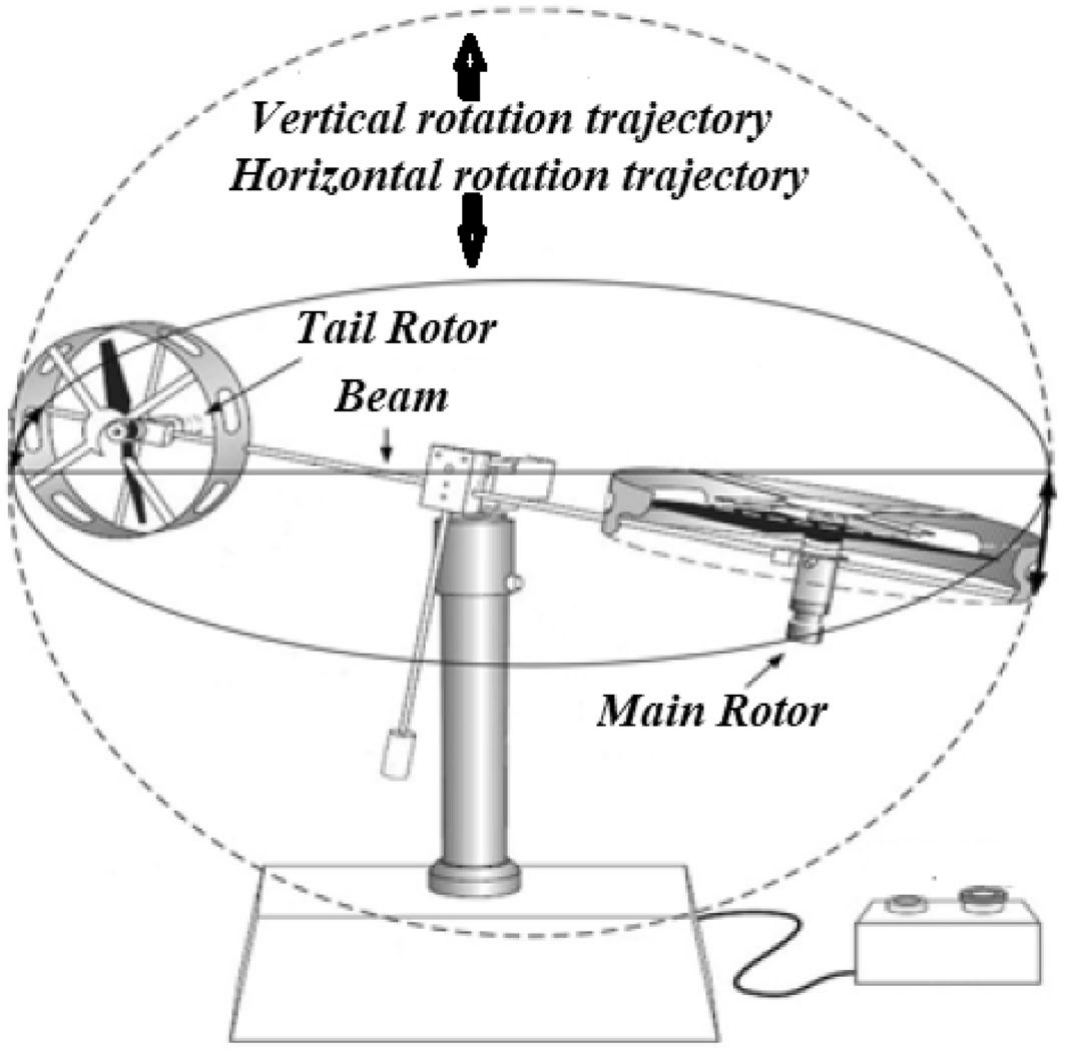
Basic schematic sketch of TRMS^13^.

Before understanding the mathematical modelling, we have to understand all varying parameters and controlling outputs of the TRMS. The TRMS is a lab apparatus provide the understanding of the flight control of helicopters^5^. The considered system has two rotors as shown in Fig. 1 and their design is most important because different forces are affecting the movement of propellers. These forces are gravitational force, propulsive force, centrifugal force, frictional force and disturbance torque. To overcome the effects of these forces we provide control input through motors. Understanding the mathematical assumptions, which are taken to understand and simplify the mathematical model. All non-linear squared terms in mathematical equations are linearized by NDI process. Two degree of freedom for the TRMS is allowed directions to tackle it. These two free movements are horizontal plane and azimuthal plane which are derived in the model:

1 $$\begin{aligned}&\frac{{d{\dot{\theta }} }}{{dt}} = \frac{{{a_1}}}{{{I_1}}}\tau _1^2 + \frac{{{b_1}}}{{{I_1}}}{\tau _1} - \frac{{{M_g}}}{{{I_1}}}\;sin\left( \theta \right) + \frac{{0.0326}}{{2{I_1}}}sin\left( {2\theta } \right) {{\dot{\varphi }} ^2} - \frac{{{B_{1\theta }}}}{{{I_1}}}\theta - \frac{{{k_{gy}}}}{{{I_1}}}cos\left( \theta \right) \mathop {{\dot{\varphi }} \;} \left( {{a_1}\tau _{1^2} + {b_1}{\tau _1}} \right) \end{aligned}$$

2 $$\begin{aligned}&\frac{{d\;{\dot{\varphi }} }}{{dt}} = \frac{{{a_2}}}{{{I_2}}}\tau _2^2 + \frac{{{b_2}}}{{{I_2}}}{\tau _2} - \frac{{{B_{1\varphi }}}}{{{I_2}}}{\dot{\varphi }} - \frac{{{k_c}}}{{{I_2}}}1.75\;\;\mathop {({a_1}}\tau _1^2 + {b_1}{\tau _1}). \end{aligned}$$

Similar momentum equations are also obtained according to the principle of momentum conservation for the rotor. Differential equations for both rotors derived here respectively as:

3 $$\begin{aligned} {{\dot{\tau }} _1} = \frac{{{T_{10}}}}{{{T_{11}}}}{\tau _1} + \frac{{{k_1}}}{{{T_{11}}}}{u_1}. \end{aligned}$$

For tail motor:

4 $$\begin{aligned} {{\dot{\tau }} _2} = \frac{{{T_{20}}}}{{{T_{21}}}}{\tau _2} + \frac{{{k_2}}}{{{T_{21}}}}{u_2}, \end{aligned}$$

where $k_1$ and $k_2$ are the motor gain, $T_{10}$, $T_{11}$ and $T_{20}, T_{22}$ are the motor parameters. $\tau _1,\tau _2$ are rotors momentum, $u_\theta ,u_\phi$ are control actions of vertical plane and horizontal plane respectively. All specific values of parameters are explained with their units in a Table 1.

## Nonlinear dynamic inversion

The feedback linearization control is also known as the NDI control which establishes a supporting platform for the linear control. Basic idea to embed this strategy is the cancellation of nonlinear terms as well as having a simplified mathematical model. The state vectors are given by:

5 $$\begin{aligned}&\dot{x}\left( t \right) = Ax\left( t \right) + Bu\left( t \right) , \end{aligned}$$

6 $$\begin{aligned}&y\left( t \right) = Cx\left( t \right) , \end{aligned}$$

here $x \in R$ (real number) of states, $u \in R$ represents the input signal and $y \in R$ measured output. The state vectors of the TRMS given below as:

7 $$\begin{aligned}&x = {\left[ {\theta \;\;\;\;\;\;\;\;\;{\dot{\theta }} \;\;\;\;\;\;\;\;\varphi \;\;\;\;\;\;\;\;{\dot{\varphi }} } \right] ^T}, \end{aligned}$$

8 $$\begin{aligned}&y = {\left[ {\theta \;\;\;\;\;\varphi \;\;\;} \right] ^T}, \end{aligned}$$

where $\theta$ is Pitch (elevation) angle, $\phi$ is yaw (azimuth) angle, $\tau _1$is momentum of main rotor and $\tau _2$ represents the momentum of tail rotor. NDI used to simplify the system by taking some simple mathematical operations. States of system linearized at origin, $x(t)=x(0)$:

9 $$\begin{aligned}&J = \frac{{\delta f\left( x \right) }}{{\delta x}}|{_{x = 0}}, \end{aligned}$$

10 $$\begin{aligned}&J = \left[ \begin{array}{*{20}{c}} {\partial {{\mathrm{f}}_1}}/ {{\partial {\mathrm{{x}}_1}}}&{} \cdots &{}{{{{\partial {\mathrm{{f}}_1}}} / {{\partial {\mathrm{{x}}_6}}}}}\\ \vdots &{} \ddots &{} \vdots \\ {{{{\partial {\mathrm{{f}}_6}}} / {{\partial {\mathrm{{x}}_1}}}}}&{} \cdots &{}{{{{\partial {\mathrm{{f}}_6}}} / {{\partial {\mathrm{{x}}_6}}}}} \end{array} \right] . \end{aligned}$$

Some mathematical operations applied to get simplified matrices at origin (0, 0, 0) shown as,

11 $$\begin{aligned}&A = \left[ {\begin{array}{*{20}{c}} 0&{}1&{}0&{}0&{}0&{}0\\ { - \frac{{{M_g}}}{{{I_1}}}}&{}{ - \frac{{{B_{1\theta }}}}{{{I_1}}}}&{}0&{}0&{}{\frac{{{b_1}}}{{{I_1}}}}&{}0\\ 0&{}0&{}0&{}1&{}0&{}0\\ 0&{}0&{}0&{}{ - \frac{{{B_{1\varphi }}}}{{{I_2}}}}&{}{ - \frac{{{k_c}}}{{{I_2}}}1.75}&{}{\frac{{{b_2}}}{{{I_2}}}}\\ 0&{}0&{}0&{}0&{}{ - \frac{{{T_{10}}}}{{{T_{11}}}}}&{}0\\ 0&{}0&{}0&{}0&{}0&{}{ - \frac{{{T_{20}}}}{{{T_{21}}}}} \end{array}} \right] , \end{aligned}$$

12 $$\begin{aligned}&C = \left[ {\begin{array}{*{20}{c}} 1&{}0&{}0&{}0&{}0&{}0\\ 0&{}0&{}1&{}0&{}0&{}0 \end{array}} \right] B = \left[ {\begin{array}{*{20}{c}} 0&{}0\\ 0&{}0\\ 0&{}0\\ 0&{}0\\ {\frac{{{k_1}}}{{{T_{11}}}}}&{}0\\ 0&{}{\frac{{{k_2}}}{{{T_{21}}}}} \end{array}} \right] . \end{aligned}$$

**Table 2:** The parameters of TRMS.

Variable notation	Parametric value	Unit
$I_1$	Main rotor inertia	$6.8 \times {10^{ - 2}}$ kg m$^2$
$I_2$	Tail rotor inertia	$2 \times {10^{ - 2}}$ kg m$^2$
$a_1$	Constant	0.0135
$b_1$	Constant	0.0924
$a_2$	Constant	0.02
$b_2$	Constant	0.9
$M_g$	Gravitational momentum	0.32 Nm
${B_{1\theta }}$	Frictional parameter	$6 \times {10^{ - 3}}$ Nm s${^2}$/rad${^2}$
${B_{2\theta }}$	Frictional parameter	$1 \times {10^{ - 3}}$ Nm s${^2}$/rad${^2}$
${B_{1\varphi }}$	Frictional parameter	$1 \times {10^{ - 1}}$ Nm s/rad
${B_{2\varphi }}$	Frictional parameter	$1 \times {10^{ - 2}}$ Nm s${^2}$/rad
$k_gy$	Gyroscopic parameter	0.05 rad/s
$k_1$	Gain of main motor	1.1
$k_2$	Gain of tail motor	0.8
$T_11$	Denominator constant of main motor	1.1
$T_10$	Denominator constant of tail motor	1
$T_21$	Denominator constant of main motor	1
$T_20$	Denominator constant of tail motor	1
$k_c$	Coupling reaction gain	2

**Figure 2:**
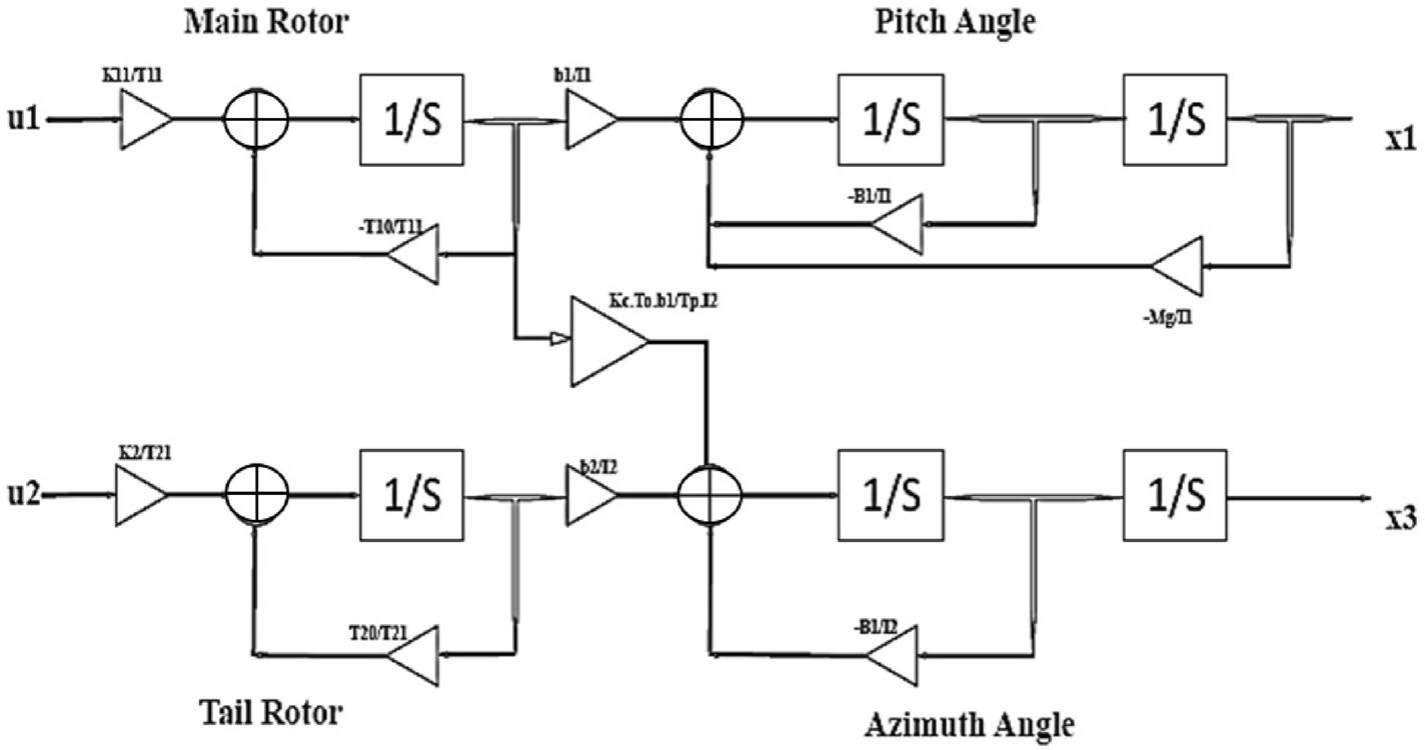
Block diagram of system.

The block diagram of the TRMS with coupling effect representation is given in Fig. 2. There are two output states called pitch angle and yaw angle. The coupling effect of main rotor on tail rotor also provided via diagram.

The NDI control strategy has some important limitations which may provide complexities during the real-time implementation of any strategy like singularity, important terms cancellation and large control tasks. Block diagram of TRMS represented in Fig. 2 and parametric values with units mentioned in Table 2.

## RGDI control design for singularity issue

To design control law for variable structure system, equations of TRMS are rearranged as:

13 $$\begin{aligned}&{\dot{x}_z} = {x_r}, \end{aligned}$$

14 $$\begin{aligned}&{\dot{x}_r} = A\left( {{x_r},t} \right) + Bu, \end{aligned}$$

here ${x_z} = \left[ {{x_1},{x_3}} \right]$ and ${x_r} = \left[ {{x_2},{x_4}} \right]$, which are dynamic states having pitch angle and yaw angle. $u = \left[ { u_\theta \left( {{w_\theta }} \right) \;,{u_\varphi }\left( {{w_\varphi }} \right) }\right]$ and $$w = \left[ {\begin{array}{*{20}{c}} {{w_\theta }}&{{w_\varphi }} \end{array}} \right]$$ are angular motion (speed) of main rotor and tail rotor respectively. The euclidean distance of a vector from origin is called euclidean norm of a linear time invariant system. In other words, magnitude of error = (true value − approximate value). In this paper, we develop estimation of state deviation calculation between control angles and desired angles known as Euclidean error norm. The attitude state deviation function $\rho$ can be defined in term of Euclidean error norm by mathematical expression given below:

15 $$\begin{aligned} \rho = {{||}}{e_z}{{||}{_w^2}} = {r_1}e_1^2 + {r_2}e_2^2 = e_z^TD\left( {{r_1},{r_2}} \right) {e_z}, \end{aligned}$$

where ${e_1} = {x_1} - {x_{1d}},\;{e_2} = {x_3} - {x_{2d}}$ and ${e_z} = \left[ {{e_1},{e_2}} \right]$. Note that the constants ${r_1}\; \& \;{r_2}$ are positive definite integers which will never be negative and ‘*D*’ is a diagonal matrix having $r_1,r_2$ as diagonal elements. The resulting equation as having the same order of differential equation and deviation function is:

16 $$\begin{aligned} {\dot{\rho }} + {c_1}\left( t \right) {\dot{\rho }} + {c_2}\left( t \right) \rho = 0, \end{aligned}$$

where $c_1$ and $c_2$ considered being tuning parameters to obtain asymptotic convergence of system dynamics^28^. The time derivatives of constraint dynamics are computed as:

17 $$\begin{aligned}&{\dot{\rho }} = 2e_z^TD\left( {{r_1},{r_2}} \right) {\dot{e}_z}, \end{aligned}$$

18 $$\begin{aligned}&{\mathop \rho \limits ^{..}}= 2e_z^TD\left( {{r_1},{r_2}} \right) \left\{ {A\left( {{x_r},t} \right) } + BU - {{\mathop {x} \limits ^{..}}_d} \right\} + 2\dot{e}_z^TD\left( {{r_1},{r_2}} \right) {\dot{e}_z}, \end{aligned}$$

here $${{\mathop {x} \limits ^{..}}_d} = \left[ {{\mathop {x} \limits ^{..}}_{1d}},{{\mathop {x} \limits ^{..}}_{2d}}\right] .$$

By putting derivatives in Eq. (14) and dynamic constraints of a system can be transformed as:

19 $$\begin{aligned} A\left( {{x_z},\;{x_r},t} \right) U = B\left( {{x_z},\;{x_r},t} \right) . \end{aligned}$$

The control function is given as:

20 $$\begin{aligned} B = -2\dot{e}_z^TD\left( {{r_1},{r_2}} \right) {\dot{e}_z}-2{c_1}e_z^TD\left( {{r_1},{r_2}} \right) {\dot{e}_z}-{c_2}e_z^TD\left( {{r_1},{r_2}} \right) {e_z}-2e_z^TD\left( {{r_1},{r_2}} \right) {A^ + }-2e_z^TD\left( {{r_1},{r_2}} \right) {{\mathop {x} \limits ^{..}}_d}. \end{aligned}$$

According to mathematical Eq. (17), system solutions are infinite which can be parameterized through a famous method known as Goreville method. By using this method:

21 $$\begin{aligned} U = {A^ + }\left( {{x_z},\;{x_r},t} \right) \;B\left( {{x_z},\;{x_r},t} \right) + P\left( {{x_z},\;{x_r},t} \right) Y, \end{aligned}$$

where *Y* represents the control vector while *P* reports about projection matrix. $A^+$ is MPGI, represented as:

22 $$\begin{aligned} {A^ + } = {A^T}\left( {{x_z},\;{x_r},t} \right) {\left\{ {A\left( {{x_z},\;{x_r},t} \right) {A^T}\left( {{x_z},\;{x_r},t} \right) } \right\} ^{ - 1}}. \end{aligned}$$

The inverse of singular matrix cannot exist because determinant of such matrix is zero. Such kind of issue in dynamic inversion considered as singularity issue. During dynamic inversion of the system matrix, it may face singularity issue with change in rank of matrix. Such condition produces discontinuity in generalized matrix function. This discontinuity becomes reason of unbounded value of matrix elements. The RGDI controller can be affected by the singularity, during the inversion process. The rank of the system matrix may be changed that generates a discontinuous behavior in the MPGI. Such kind of drawbacks can be covered by augmentation of scaling factor to expression discussed in Ref.^29^ and elaborated as:

23 $$\begin{aligned} \dot{v}\left( t \right) = - v\left( t \right) + \frac{\gamma }{{\mathrm{{||}}{e_z}\left( t \right) \mathrm{{|}}{\mathrm{{|}}^2}}},\;v\left( 0 \right) > 0, \end{aligned}$$

where ${e_z}\left( t \right) = [{e_1}\left( t \right) ,{e_2}\left( t \right) \mathrm{{]}}$. Asymptotic stability confined through the above expression and modified form of system equation of matrix is given below as a function:

24 $$\begin{aligned} A\left( {{x_z},\;{x_r},v,t} \right) = {A^T}\left( {{x_z},\;{x_r},t} \right) {\left\{ {A\left( {{x_z},\;{x_r},t} \right) {A^T}\left( {{x_z},\;{x_r},t} \right) + v\left( t \right) } \right\} ^{ - 1}}. \end{aligned}$$

The extended condition of controller input as:

25 $$\begin{aligned} {U^*} = A\left( {{x_z},\;{x_r},v,t} \right) \;B\left( {{x_z},\;{x_r},t} \right) + P\left( {{x_z},\;{x_r},t} \right) Y. \end{aligned}$$

DSGI expression is given as:

26 $$\begin{aligned} {\dot{x}_r} = {A^*}({x_r},t) + BA({x_z},{x_r},v,t)B({x_z},{x_r},t) + P({x_z},{x_r},t)Y. \end{aligned}$$

All the elements of $A\left( {{x_z},\;{x_r},v,t} \right)$ are bounded for $t \ge 0$^30^. As we have discussed the reduction of system matrix rank produce discontinuity, now to elaborate the rank of the system matrix is not going to be reduced by calculating its controllability and observability matrix to understand the stability analysis. The system under consideration is being checked by a calculation process such as controllability and observability. The controllability property of the system, coupling within the state’s and the input, involve the system matrices *A* and *B*. The $C_c$ matrix is a linear system said to be in controllable form if it is possible to find the some input *u*(*t*), and this input will transform the state’s x(to) to the origin at finite time. If there exist some input $u(t_1)$ and gives $x(t)=0$, admitted for all initial times and state’s, then it is verified for controller^31^. The controllability elaborated as:

(i) (*A*, *B*) is controllable,
(ii) The controllability matrix can be found as:
  27 $$\begin{aligned} {C_c}= [B\;AB\;{A^2}B\;{A^3}B\;{A^4}B\; \ldots \;{A^{\alpha - }}^1B], \end{aligned}$$

where $\alpha$ is positive integer which depends on the order of system matrix A and order of matrix in above equation is, $\alpha =6$. System will be considered as controllable if it’s determinant is non-zero. Matrix show the full rank property so, system is controllable. The full rank of the matrix as mentioned above, provide strong validation towards the proof of the controllable system. The calculated controllability matrix is,

28 $$\begin{aligned} {C_c} = \left[ {\begin{array}{*{20}{c}} 0&{}0&{}0&{}0&{}{0.0014}&{}0&{}{ - 0.0014}&{}0&{}{ - 0.0052}&{}0\\ 0&{}0&{}{0.0014}&{}0&{}{ - 0.0014}&{}0&{}{ - 0.0052}&{}0&{}{0.0058}&{}0\\ 0&{}0&{}0&{}0&{}{0.0016}&{}{0.0036}&{}{ - 0.0096}&{}{ - 0.0216}&{}{0.0491}&{}{0.1116}\\ 0&{}0&{}{ - 0.0016}&{}{0.0036}&{}{0.0096}&{}{0.0216}&{}{0.0491}&{}{0.1116}&{}{0.2468}&{}{0.5616}\\ 1&{}0&{}{0.0009}&{}0&{}{0.0008}&{}0&{}{0.0008}&{}0&{}{0.0007}&{}0\\ 0&{}{0.8}&{}0&{}{0.0008}&{}0&{}{0.0008}&{}0&{}{0.0008}&{}0&{}{0.0008} \end{array}} \right] . \end{aligned}$$

The excellent validation of full rank system can be viewed from the above matrix which shows a full rank matrix. A system having full rank property can be verified by finding the determinant of system matrix which should not equal to zero. All states of the system converge to origin validates the system observability. The observability of the system can be verified as,

(i): (A, C) is observable,
(ii): The observability matrix can be found here:
  29 $$\begin{aligned} O_O = {[C\;CA\mathrm{{ }}C{A^2}\;C{A^3}\;C{A^4}......\mathrm{{ }}C{A^{\alpha - }}^1]^T} \end{aligned}$$

where $\alpha$ represents the order of system matrix *A* and value in above equation is, $\alpha =6$. The system will be considered as control able if it’s determinant is non-zero. The matrix show the full rank property so, system is observable. The calculated observability matrix is given below:

30 $$\begin{aligned} {O_O} = \left[ {\begin{array}{*{20}{c}} 1&{}0&{}0&{}0&{}0&{}0\\ 0&{}0&{}1&{}0&{}0&{}0\\ 0&{}1&{}0&{}0&{}0&{}0\\ 0&{}0&{}0&{}1&{}0&{}0\\ { - 4.7059}&{}{ - 0.0882}&{}0&{}{1.3588}&{}0&{}0\\ 0&{}0&{}0&{}{ - 5}&{}{1.6170}&{}{4.5}\\ {0.4151}&{}{ - 4.6534}&{}0&{}0&{}{ - 1.3543}&{}0\\ 0&{}0&{}0&{}{25}&{}{ - 9.5532}&{}{ - 27}\\ {22.1089}&{}{0.8294}&{}0&{}0&{}{ - 8.6574}&{}0\\ 0&{}0&{}0&{}{ - 125}&{}{49.1258}&{}{139}\\ { - 3.9032}&{}{22.0356}&{}0&{}0&{}{5.8103}&{}0\\ 0&{}0&{}0&{}{663}&{}{ - 246}&{}{ - 702} \end{array}} \right] . \end{aligned}$$

Full rank property can be verified from the above matrix and ensure the observability of the system. Stability analysis provides a strong platform to design a suitable controller. Next section elaborates the controller constraints according to system dynamics.

## Design of SMC and simulations

The concept of SMC is based on VSS control theory and work on the principle that controller structure will change continuously with variation in the state variables to keep the system states in sliding mode.

**Figure 3:**
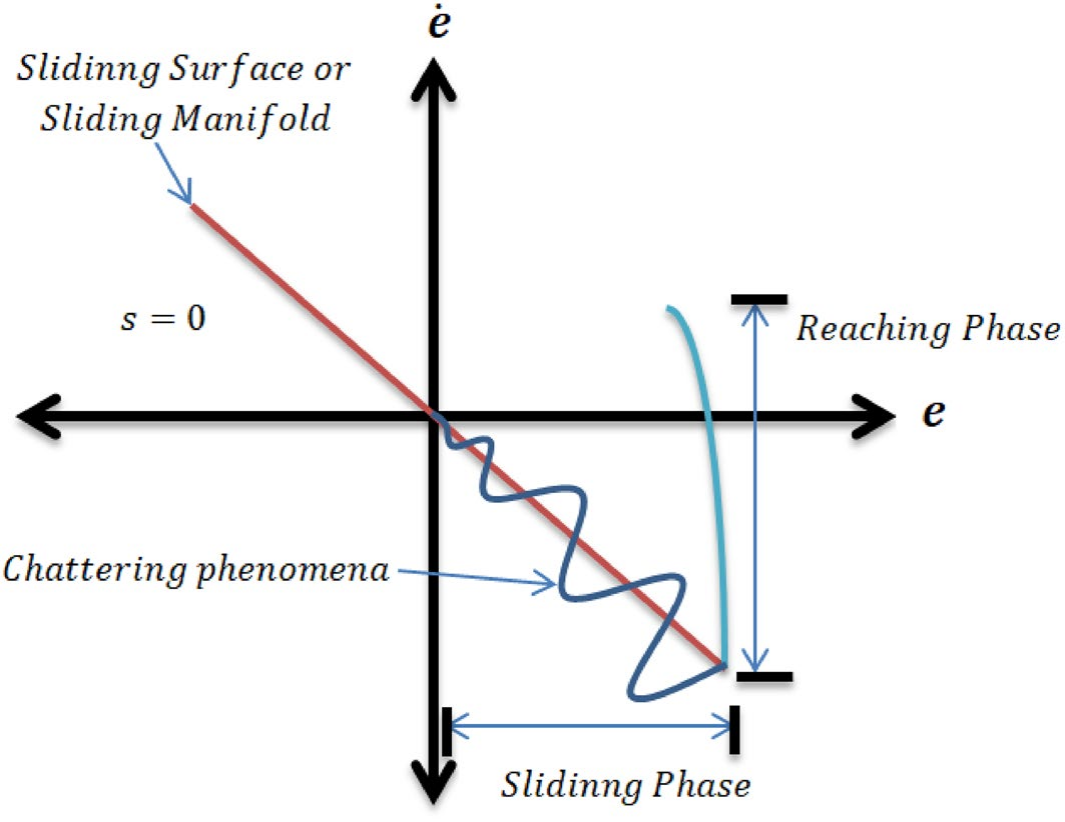
Chattering phenomena.

**Figure 4:**
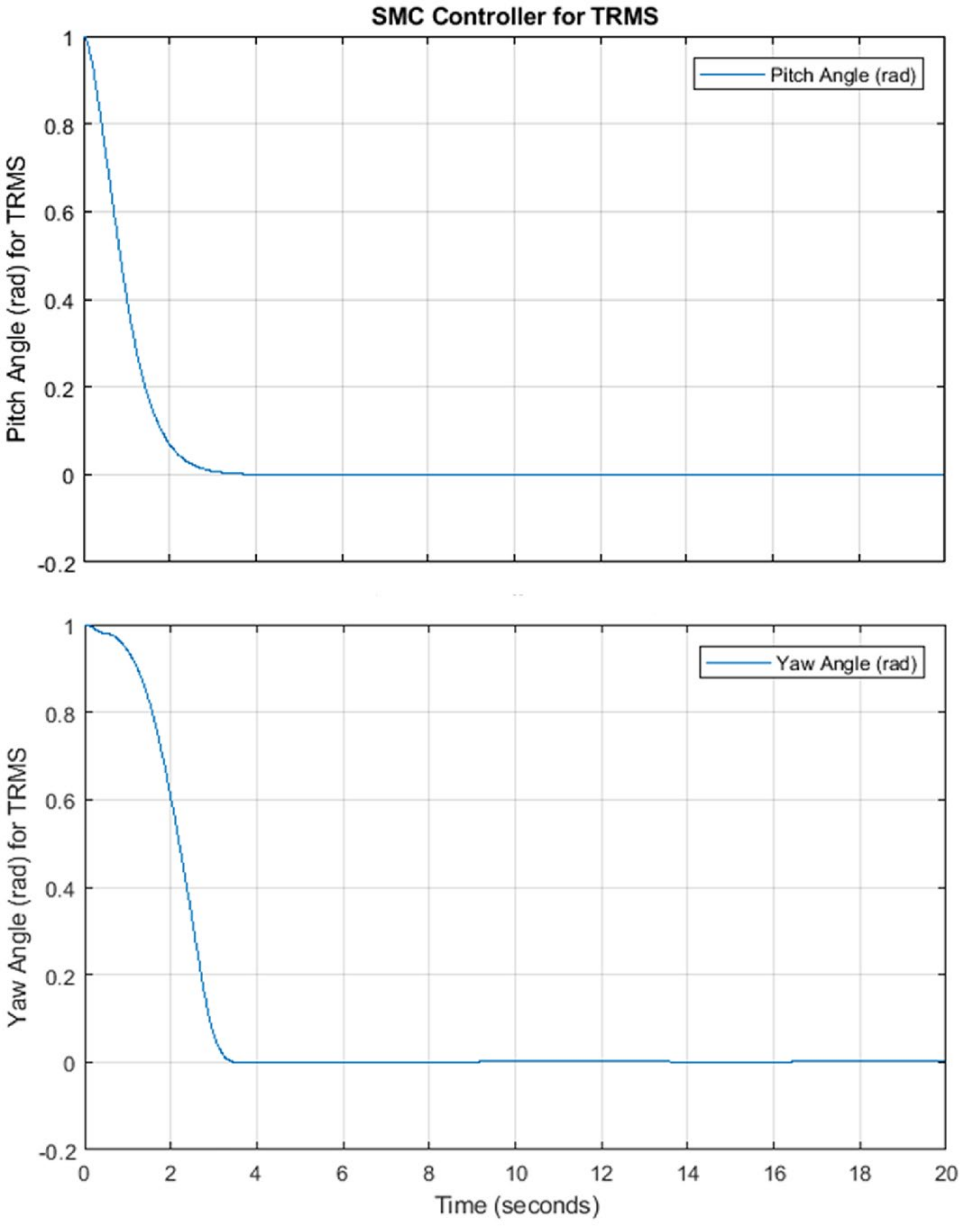
Pitch and Yaw Angle of TRMS using SMC.

The SMC tends to modify the system dynamics by applying switching control of high frequency. The basic design of SMC can be elaborated in two steps. First we have to choose the sliding surface according to the order of considered system.

**1st Step**

The sliding manifold (surface) for this system can be selected as:

31 $$\begin{aligned} S\left( t \right) = {\left( {\frac{d}{{{d_t}}} + \lambda } \right) ^n}\;\mathop \smallint \nolimits _0^t e\left( t \right) \;{d_t}, \end{aligned}$$

where *S*(*t*) represents the sliding surface with respect to time and *e*(*t*) is tracking output.

**2nd Step**

**Figure 5:**
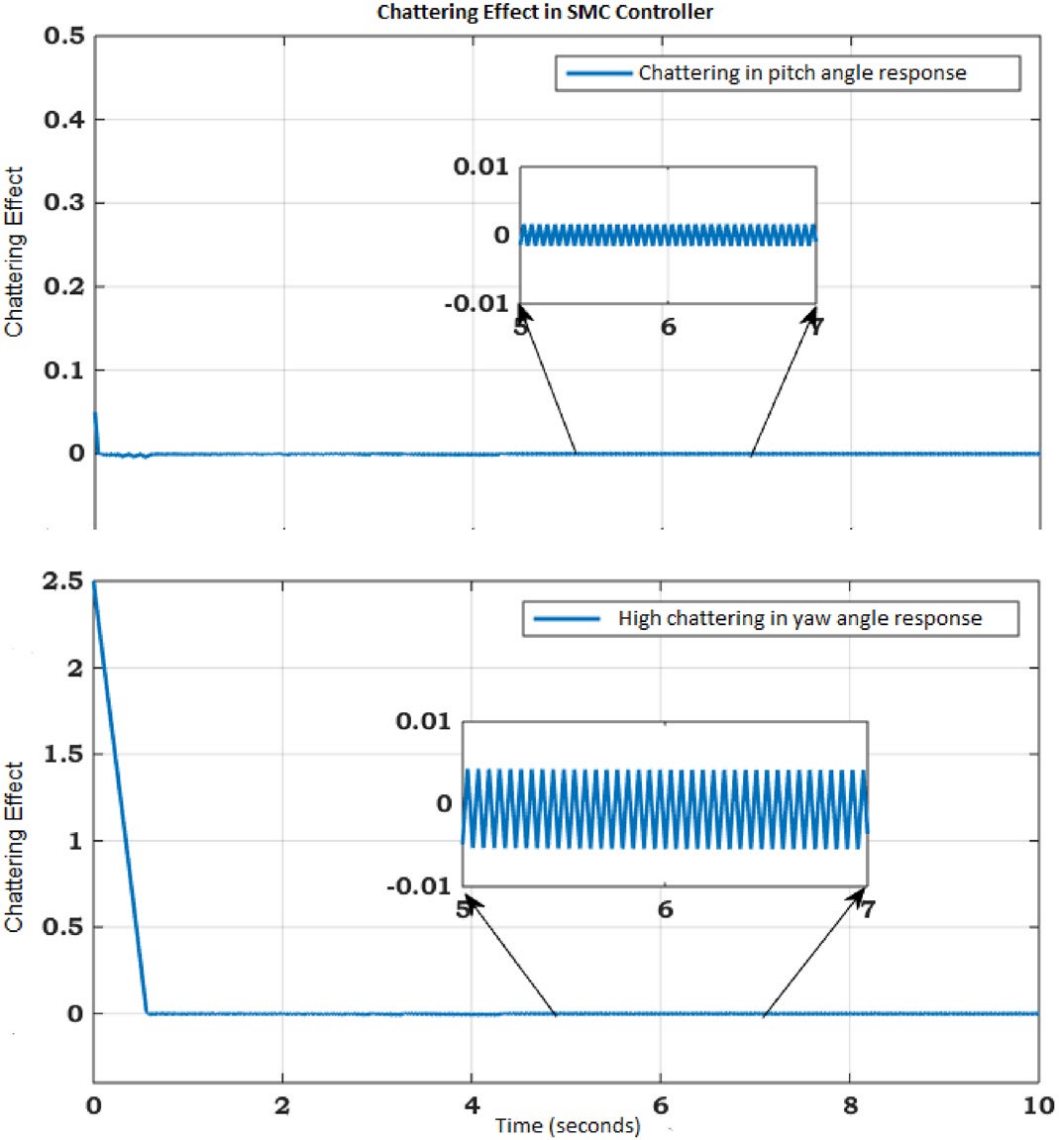
Chattering in control input for Pitch and Yaw Angle.

When the sliding surface is selected, we must focus on the control law. The control law drives the controlled variables to its reference value. The mathematical expression for control law can be defined as:

32 $$\begin{aligned} u = {u_{eq}} + {u_{dis}}, \end{aligned}$$

while

33 $$\begin{aligned} {u_{dis}} = - {k_1}sign\left( S \right) , \end{aligned}$$

here $u_{eq}$ represent equivalent controller and $k_1$ constant. The controlled system trajectory slide along the manifold by the action of multiple control structures and will follow the switching condition. In this way, the controlled system ultimate follow the trajectory towards convergence as shown in Fig. 3. It has been observed that during SMC, the system structure is defined by switching functions (*x*), where *x* is either a scalar or vector. The switching surface represented by $s(x)=0$, is a line on the phase plane. The lyapunov function is a platform to analyze the stability of the nonlinear system based on ordinary differential equations (ODE) theory. The ODE class theory validates the stability of the system by calculation of system Lyapunov function that must be negative definite. This sufficient condition ensures the asymptotic stability of the nonlinear system. We don’t have the proper strategy to construct function for ODEs^27^. In practice, it has been observed that the sliding motion exists in the area around the sliding surface just like switching phenomena of frequency.The nonlinear behavior of the system will try to deviate from the sliding surface but the controller will enforce to follow line till system converged at the origin, known as the boundary layer. The SMC simulation results show the satisfactory convergence for pitch angle and yaw angle. The pitch angle must be converged to zero or stabilized before the yaw angle in Fig. 4. The SMC chattering pattern for such kind of systems can never be suitable because it will be dangerous for actuators as in Fig. 5. The rapid and sudden changes in voltage pattern required highly optimized power supplier. The TRMS output required smooth convergence with regular voltage pattern can be optimized by the optimization method.

## RGDI based ${H_\infty }$ optimization and simulations

**Figure 6:**
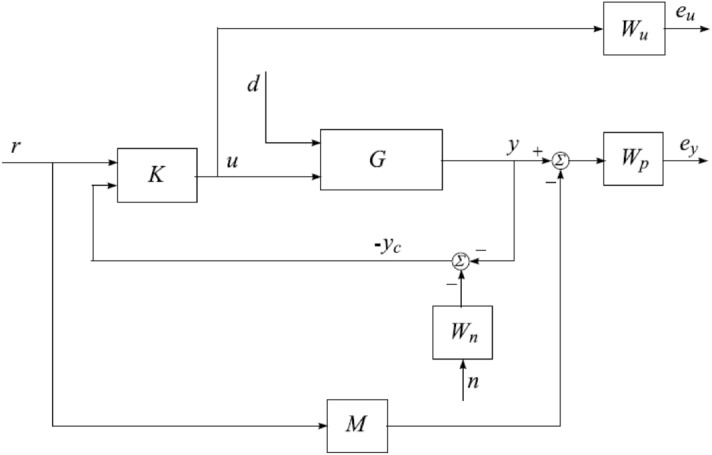
Closed-loop system with performance requirements.

**Figure 7:**
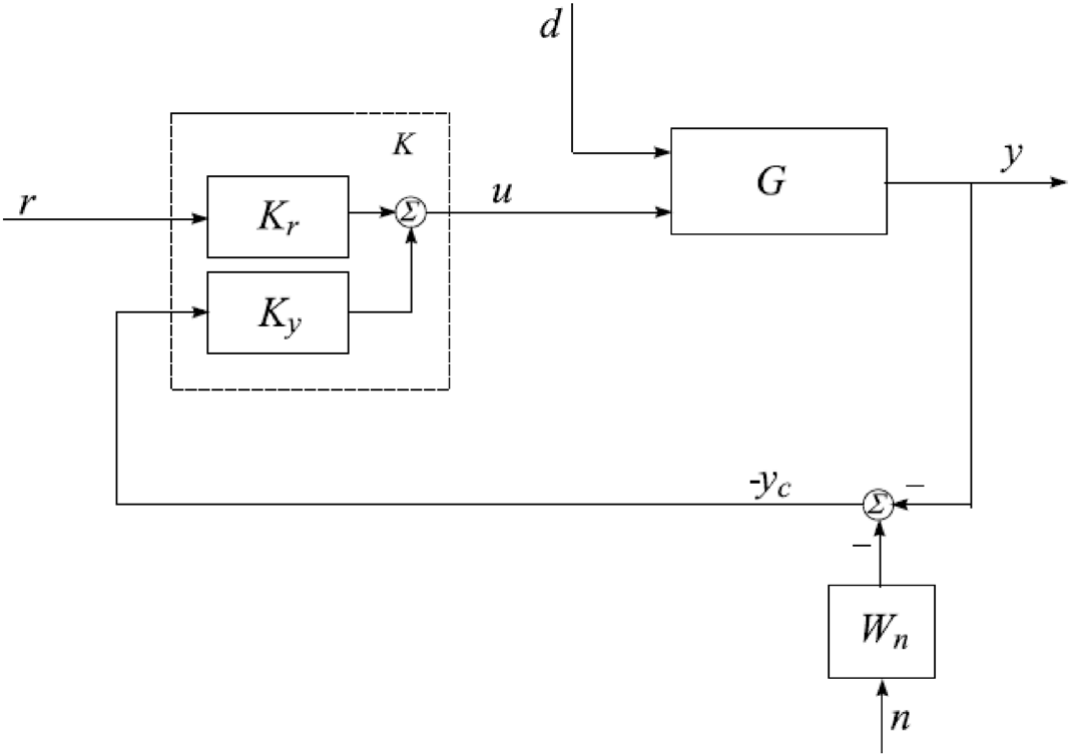
Block diagram of H-infinity control.

Some important symbolic representation is being addressed here to make reader friendly. Now, overall system represented by “$$G = \left[ {\begin{array}{*{20}{c}} {{G_d}}&{{G_u}} \end{array}} \right]$$” while $G_d$ represents the disturbance of plant as matrix and $G_u$ shows the transfer matrix of control signal. The specification design for the control system is tracking of the desired signal. The output of the designed control system should follow the preselected signal. The un-certain plant has some basic requirements in the presence of perturbations like internal and external disturbance as shown in Fig. 6. We represent weighting functions $W_p$ and $W_u$, which are reflecting the trade-off among characteristics of *and*/*or* for signals.The output required control task with performance and stability requirements represented via mathematical equation given below:

34 $$\begin{aligned} u = \left[ {\begin{array}{*{20}{c}} {{K_r}}&{{K_y}} \end{array}} \right] {\left[ {\begin{array}{*{20}{c}} r&{ - {y_c}} \end{array}} \right] ^T} = {K_r}r - {K_y}{y_c}, \end{aligned}$$

where $K_y$ represents the feedback matrix function and $K_r$ is transfer function matrix of pre-filter. The closed-loop model (uncertain TRMS) shown in Fig. 7, represents the controller feedback response, performance requirement and disturbance matrix of noise function. Different variables like *r*, *d* and *n* represents the reference input, input disturbance and noise respectively. The output angles as yaw angle $\alpha _h$ and pitch angle $\alpha _v$ are required to control (measure) under all kind of perturbations (noise, parametric). The output tracking control signals $e_y$ and $e_u$ are error tracking signals. The output feedback vector $y_c=y+W_{n_n}$, is vector matrix having measured noise *n* and $W_n$ filter for noise shaping. The following weighted system required error tracking output $(e_y\; and\; e_u)$ equation must satisfy the condition:

35 $$\begin{aligned} \left[ {\begin{array}{*{20}{c}} {{e_y}}\\ {{e_u}} \end{array}} \right] = \left[ {\begin{array}{*{20}{c}} {{W_p}\left( {{S_o}{G_u}{K_r} - M} \right) }&{}{{W_p}{S_o}{G_d}}&{}{ - {W_p}{S_o}{G_u}{K_y}{W_n}}\\ {{W_u}{S_i}{K_r}}&{}{ - {W_u}{S_i}{K_y}{G_d}}&{}{ - {W_u}{S_i}{K_y}{W_n}} \end{array}} \right] \left[ {\begin{array}{*{20}{c}} {\begin{array}{*{20}{c}} r\\ d \end{array}}\\ n \end{array}} \right] , \end{aligned}$$

while $S_i=(I+K_y G_u )-1$ and $S_o=(I+G_u K_y )-1$ shows input, output sensitivity matrix function respectively.

**Table 3:** Weighting function.

Functions	Description
$W_p (S_o G_u K_r-M)$	Weighting difference
$W_p S_o G_d$	Weighted sensitivity to disturbance
${W_p}{S_o}{G_u}{K_y}{W_n}$	Weighted sensitivity to noise
$W_u S_i K_r$	Weighted control action due to reference
$W_u S_i K_y G_d$	Weighted control action due to disturbance
$W_u S_i K_y W_n$	Weighted control action due to noise

The performance criterion requires the transfer function matrix from the exogenous input signals *r*, *d* and *n* to the output signals $e_y$ and $e_u$ to be small , for all possible uncertain plant model *G*. The transfer function matrices $W_p$ and $W_u$ are used to reflect the relative importance of different frequency ranges for which the performance requirements should be fulfilled. The six transfer function matrices which constitute the transfer function matrix between the inputs and outputs of the extended system are described in Table 3. The controller design task is to regulate required output:

36 $$\begin{aligned} K = \left[ {{K_r}\;\;\;\;\;\;\;\;\;\;\;\;{K_y}} \right] . \end{aligned}$$

That must elaborate and satisfied the enlisted properties under perturbations. The robust stability under perturbations must meet the required response by satisfying closed loop nominal performance and robust response conditions. The condition for nominal performance:

37 $$\begin{aligned} {\left[ {\begin{array}{*{20}{c}} {{W_p}\left( {{S_{o,nom}}{G_{u,nom}}{K_r} - M} \right) }&{}{{W_p}{S_{o,nom}}{G_{d,nom}}}&{}{ - {W_p}{S_{o,nom}}{G_{u,nom}}{K_y}{W_n}}\\ {{W_u}{S_{i,nom}}{K_r}}&{}{ - {W_u}{S_{i,nom}}{K_y}{G_{d,nom}}}&{}{ - {W_u}{S_{i,nom}}{K_y}{W_n}} \end{array}} \right] _\infty } < 1. \end{aligned}$$

The condition for robust performance:

38 $$\begin{aligned} {\left[ {\begin{array}{*{20}{c}} {{W_p}\left( {{S_o}{G_u}{K_r} - M} \right) }&{}{{W_p}{S_o}{G_d}}&{}{ - {W_p}{S_o}{G_u}{K_y}{W_n}}\\ {{W_u}{S_i}{K_r}}&{}{ - {W_u}{S_i}{K_y}{G_d}}&{}{ - {W_u}{S_i}{K_y}{W_n}} \end{array}} \right] _\infty } < 1. \end{aligned}$$

Above conditions must be satisfied for *G*. The specification design for the control system is tracking of the desired signal. The output of the designed control system should follow the preselected signal. According to the behavior of the nonlinear system, we must have a 2DOF ${H_\infty }$ controller rather than other controllers as discussed in Ref.^32^. By this technique, two controllers are designed, one for the robust stability, the internal stability and the rejection of disturbance, while the other controller design for the minimization of error between the reference signal and the actual response of the model.

The functions $K_r$ and $K_y$ are transfer function of system matrix which can be easily obtained. The system is described as:

39 $$\begin{aligned} \left[ {\frac{{\begin{array}{*{20}{c}} {{z_1}}\\ {{z_2}} \end{array}}}{{\begin{array}{*{20}{c}} {{e_1}}\\ {{e_2}} \end{array}\;\;}}} \right] \mathrm{{\;}} = \mathrm{{\;\;\;}}\left[ {\frac{{\begin{array}{*{20}{c}} { - {W_p}M}&{}{{W_p}}\\ 0&{}0 \end{array}\;\mathrm{{|}}\;\begin{array}{*{20}{c}} {{W_p}G}\\ {{W_u}} \end{array}}}{{\;\begin{array}{*{20}{c}} I&{}{0\;\;\;\;\;\;}\\ 0&{}{I\;\;\;\;\;\;} \end{array}\mathrm{{|}}\;\;\;\begin{array}{*{20}{c}} 0\\ G \end{array}}}} \right] \left[ {\frac{{\begin{array}{*{20}{c}} r\\ d \end{array}}}{{\;\begin{array}{*{20}{c}} u\\ u \end{array}\;}}} \right] . \end{aligned}$$

The closed loop system transfer function can be obtained as:

40 $$\begin{aligned} {T_{zw}} = \left[ {\begin{array}{*{20}{c}} {{W_p}\left( {{S_o}{G_u}{K_r} - M} \right) }&{}{{W_p}{S_o}}\\ {{W_u}{S_i}{K_r}}&{}{{W_p}{K_y}{S_o}} \end{array}} \right] . \end{aligned}$$

The task is to minimize the cost function value $({H_\infty }norm)$ of $T_zw$ and get stable gain. The weighting functions are selected to regulate system required output response as, the weighting function matrix chooses are given below:

41 $$\begin{aligned} {w_m} = \left[ {\begin{array}{*{20}{c}} {{w_{m_{11}}}}&{}{{w_{m_{12}}}}\\ {{w_{m_{21}}}}&{}{{w_{m_{22}}}} \end{array}} \right] . \end{aligned}$$

This is a model matrix for ideal model.

42 $$\begin{aligned} {w_p} = \left[ {\begin{array}{*{20}{c}} {{w_{p_{11}}}}&{}{{w_{p_{12}}}}\\ {{w_{p_{21}}}}&{}{{w_{p_{22}}}} \end{array}} \right] . \end{aligned}$$

This is a matrix for performances.

43 $$\begin{aligned} {w_u} = \left[ {\begin{array}{*{20}{c}} {{w_{u_1}}}&{}{{0}}\\ {{0}}&{}{{w_{u_2}}} \end{array}} \right] . \end{aligned}$$

This is a matrix for control action.

44 $$\begin{aligned} {w_n} = \left[ {\begin{array}{*{20}{c}} {{w_{n_1}}}&{}{{0}}\\ {{0}}&{}{{w_{n_2}}} \end{array}} \right] . \end{aligned}$$

**Figure 8:**
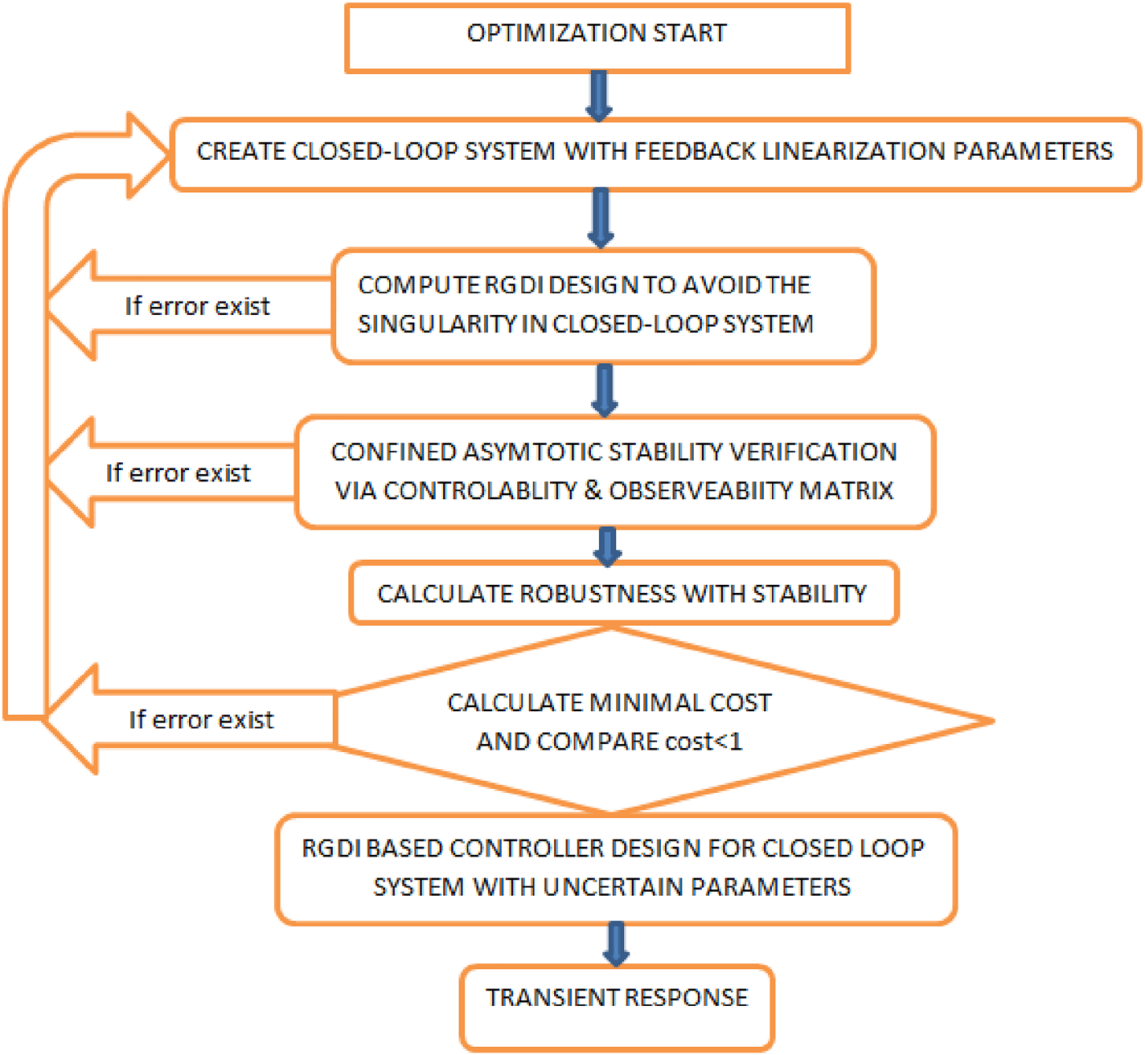
Flow chart of optimization design steps.

This is a matrix for sensor noise. We aim to minimize the error between the output and the desired signal, to obtain the good robustness performance and the stability performance. The controller design flowchart given in Fig. 8. The procedure for the controller is given below:

- Create the uncertain parameters of the system.
- Create the open-loop model by “sysic”.
- Design the weighting matrices by several iterations.
- Create the closed-loop plant including the weighting matrices.
- the stability and robustness performance against the weighting matrices. Conditions
- Lower bound $>1$ than system ensure robust stability against uncertainties.
- If the upper bound $< 1$ than the system will not ensure the robust stability against uncertainties.
- If the lower bound $<1$ and upper bound $>1$ it would be an impossible case to conclude decision about the robust response.

Let the lower bound is 1 and upper bound is 1.3, it means that the system gives stability and the robustness performance against the parametric and model uncertainties. Check the gamma values if the value less than one than the nominal performance can be achieved. Then design the ${H_\infty }$ controller for the system and the check the closed-loop transient responses. To design the open loop model we use five functions of “*sysic*” for main rotor, pitch angle, tail rotor, azimuth angle and last for the system interconnections.

$$\mathrm{{systemnames = main \; rotor \; pitch \; angle \; tail \; rotor \; azimuth \; angle;}}$$

$$inputvar = [dist2;control2]$$

$$outputvar = \mathrm{{[main \; rotor; pitch \; angle; tail \; rotor; azimuth \; angle]}}$$

$$input \; to \; main \; rotor = \mathrm{{[control(1)]}}$$

$$input \; to \; pitch \; angle = \mathrm{{[dist(1); main \; rotor; control(2)]}}$$

$$input \; to \; tail \; rotor = [control(2)]$$

$$input \; to \; azimuth \; angle = [dist(2); main \; rotor; tail \; rotor; control (1)]$$

$$\mathrm{{G = sysic}}$$

**Figure 9:**
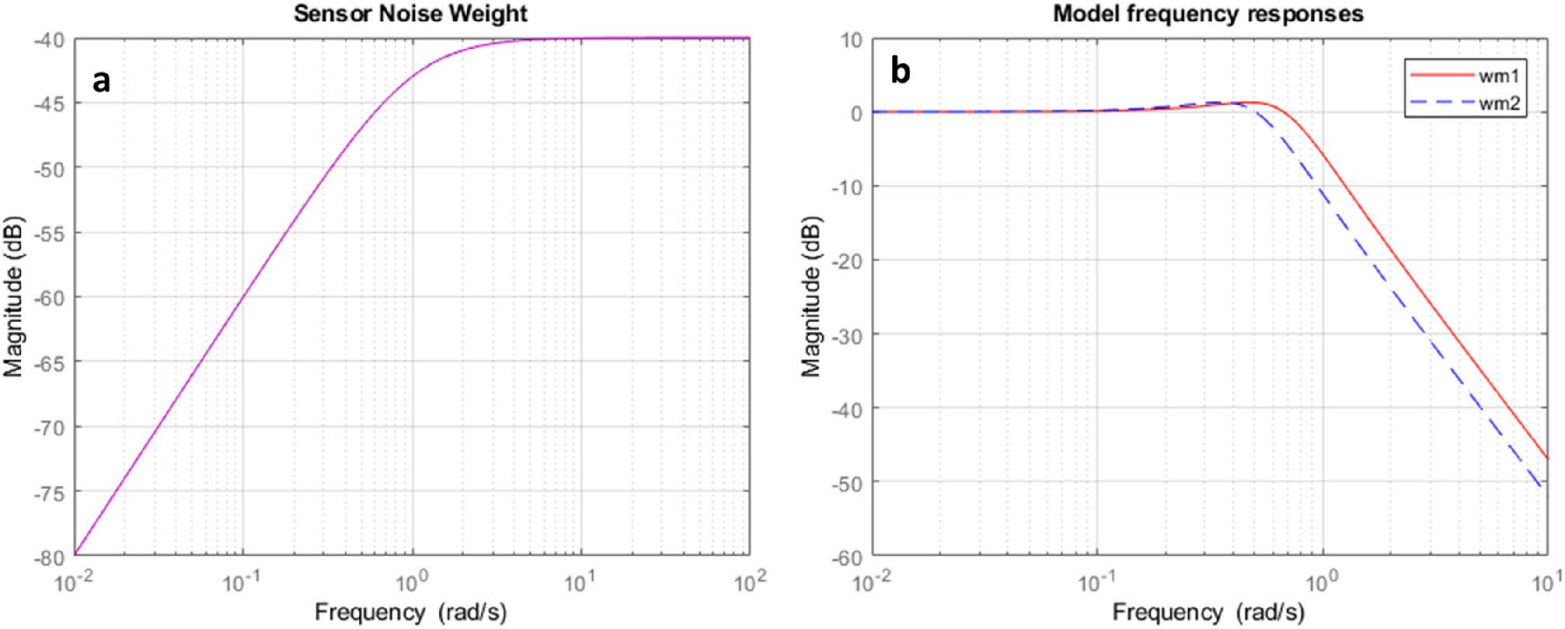
Sensor noise and model frequency response.

The closed-loop plant interconnections of the model including the weighting matrix can be created as and the singular value plot shown in simulation result. Gamma values achieved using ${H_\infty }$ controller. $\gamma = 0.1006$, this value is less than one so, the stability and robustness performance can be achieved. The range of gamma iteration considered from 0.1 to 10 with tolerance 0.001, the result shows that for which values of gamma are accepted and which are not. The noise weight function and obtained model frequency response of TRMS shows in Fig. 9a,b respectively.

**Figure 10:**
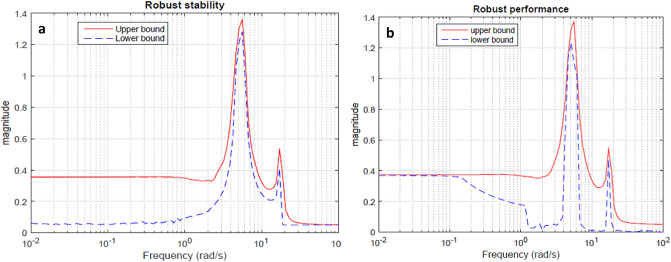
Robust stability and robust performance.

The simulations are carried out to test the robustness of implemented techniques concerning the matched and mismatched perturbations. The perturbed model of the TRMS is used to carry out simulations. The robust stability and robust performance represented via simulation results in Fig. 10a,b respectively.

**Figure 11:**
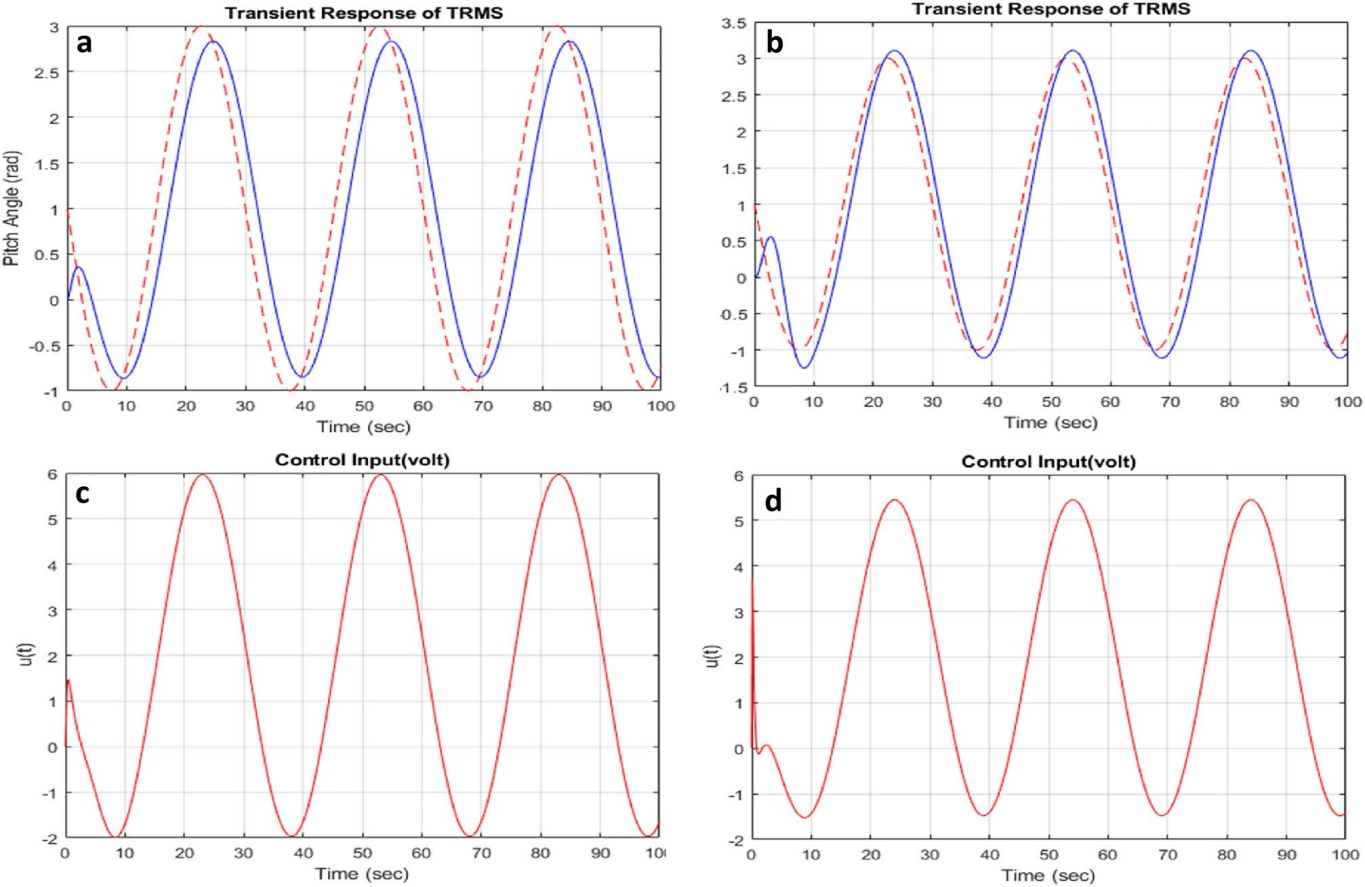
Angles response with control input response under sine wave.

The simulation results in the presence of disturbance signal provide a validation towards the robust response of optimization strategy. A sinusoidal signal is provided as input signal to ensure the tracking performance of the optimization method which is being subjected by some nonlinear disturbances. The pitch angle and yaw angle tracking performance of the desired output can be examined from Fig. 11a,b. The red curves represent the reference input of both angles (pitch and yaw)and blue curve shows the tracking output of the pitch and yaw angles of the TRMS. The conditions for robust stability and robust performance already explained in controller design section. The control action response can also be visualized from results in Fig. 11c,d, which represents the smooth convergence of the system towards stability.

## Experimental setup and system connections

**Figure 12:**
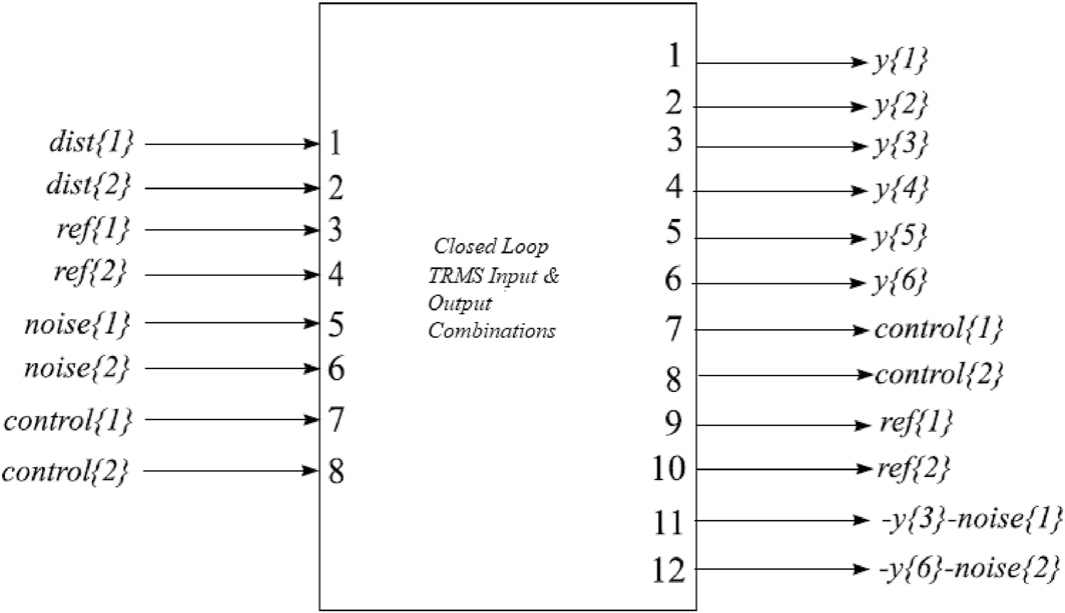
System internal structure of closed loop TRMS.

In this section, we elaborate concept of real-time implementation and system interconnections through system integrated circuits. A brief discussion is also provided concerning the response of the valid output results. The internal structure of the system is also labeled with ports, to understand implementation more precisely for the reader.The schematic diagram of the closed-loop system with important variables description elaborated in Fig. 12 and the number of input–output ports also provided to understand internal structure easily.

**Figure 13:**
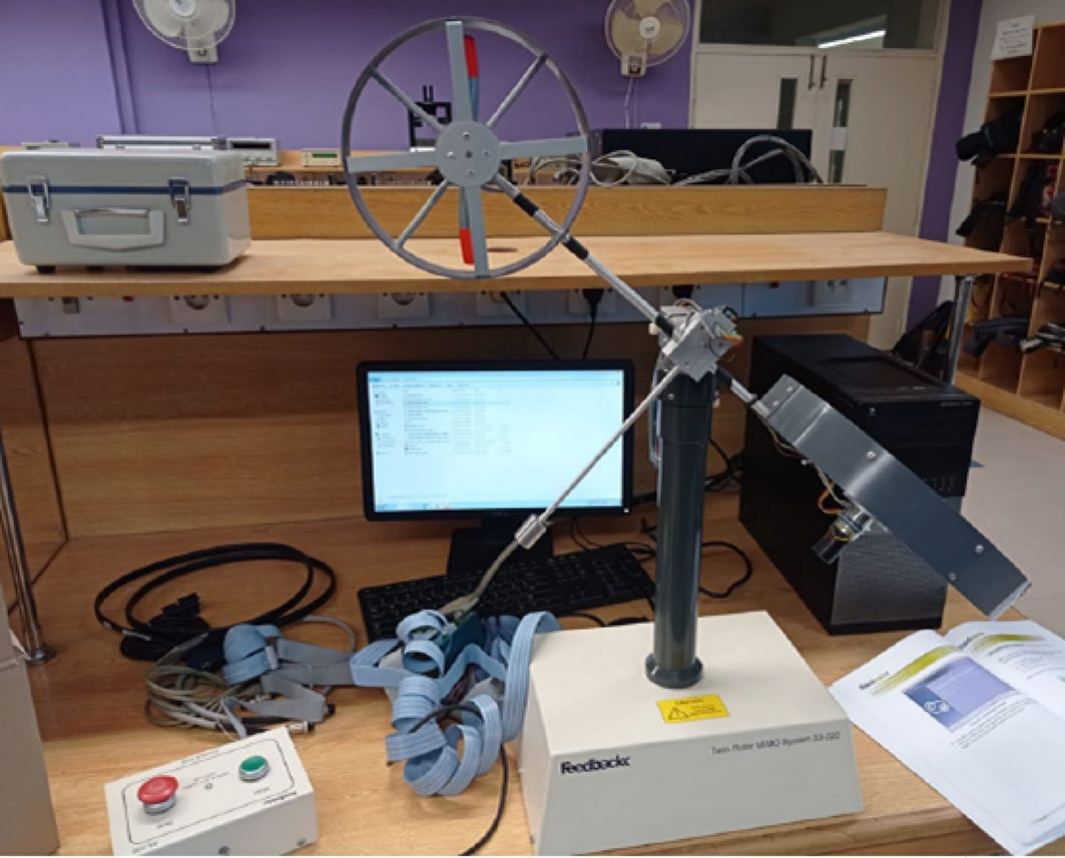
Experimental apparatus (Prototype).

The real time implementation of the prototype can be viewed in Fig. 13. The laboratory setup with computer and power supply are three main components of experimental apparatus.

**Figure 14:**
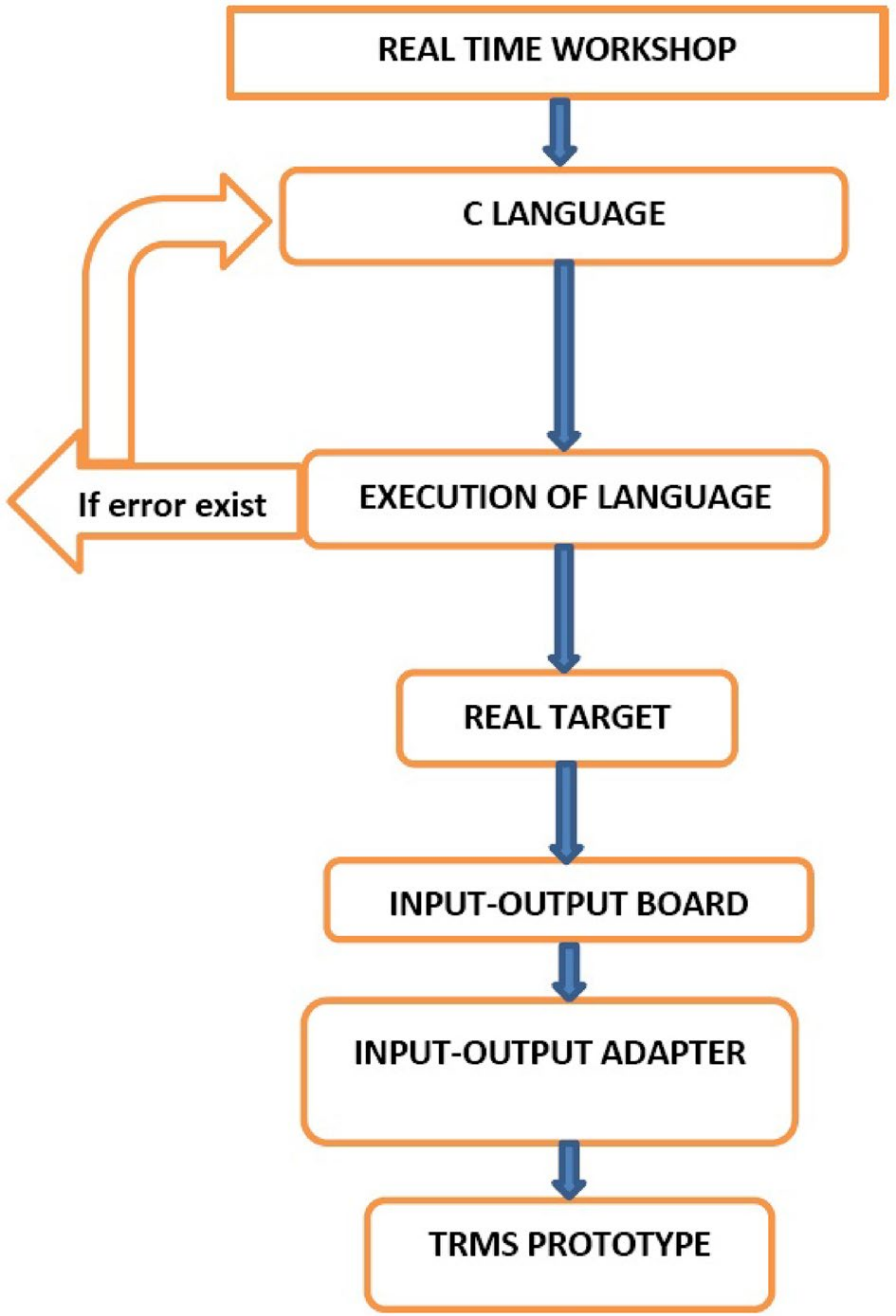
Flow chart TRMS laboratory setup implementation.

The RGDI based ${H_\infty }$ optimization is done for the several performances weighting matrix (tuning factor). The control action for both rotors via optimization control elaborated through simulation response. The application of the controller allows the unwanted signals to reject perturbations like parametric measurements, disturbance torque, thrust, and external uncertainty. To validate the excellent robust performance, the model being disturbed through 10*percent* parametric uncertainty with disturbance noise signals on both rotors would be a worse case of robustness in real-time implementation.

Disturbance (1) = 0.2 and disturbance (2) = 0.2, white noise (1) = 0.1 and the noise matrix as $${W_n} = \left[ {\begin{array}{*{20}{l}} {{w_n}\left( s \right) }&{}0\\ 0&{}{{w_n}\left( s \right) } \end{array}} \right]$$ and transfer function is ${w_n} = {10^{ - 2}}\frac{s}{{s + 1}}$ with unit radian. All mentioned perturbations (disturbances) are applied separately to each rotor of highly coupled system to verify the worth of simulation results. The real-time implementation with the help of a robust designed controller validates the controller worth under disturbances (noise signal, un-modeled states, parametric, coupling effect).

**Figure 15:**
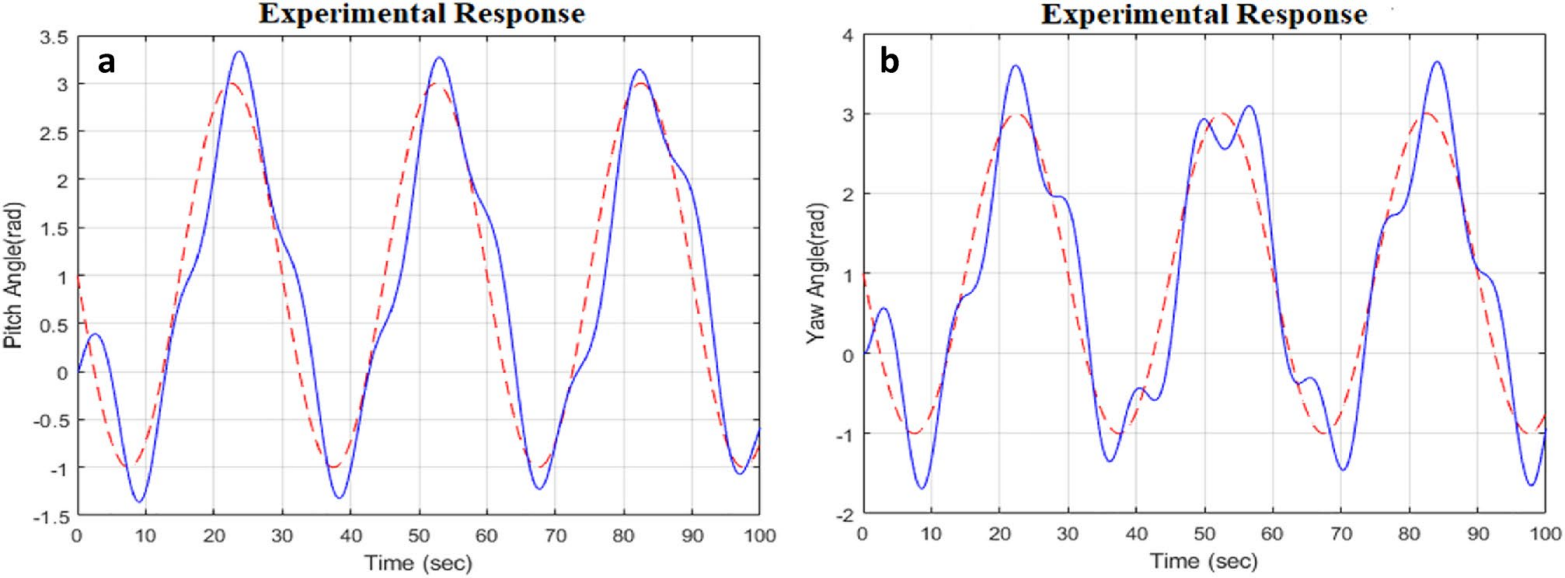
Pitch and yaw angle experimental response.

The experimental processing of the TRMS with all required steps is mentioned in the Fig. 14 to understand the implementation. The limited varying speed provided to validate the system robust response with stability credibility of the controller. The experimental output response of the pitch angle with their control action shown in Fig. 15a,b that validates the system’s sharp response towards convergence within limited variationgh attenuation during tracking is the coupling effect generated by the main rotor as we in the range of input control voltage. The small attenuation in the amplitude response as compared to yaw angle response is due to highly nonlinear behavior and perturbations (noise, parametric). Comparatively yaw angle shows more overshoot in its amplitude response. Similarly, a sharp variation in the control action of the yaw angle can be observed. A high level of noise (disturbance), causes a serious problem with the actuators and input control signal as an error. To get actual actuator input the first-order filter is based on the butterworth filter used.

## Conclusion

This work is an attempt to understand the design of robust optimization technique based on generalized dynamic inversion for a highly nonlinear, cross-coupled MIMO system. In the optimization strategy, time-varying dynamic constraints are designed and output states are being tracked by the reference trajectories. Sinusoidal reference tracking of states ensures robustness and stability validation against considered uncertainties. The behavior of the system demonstrates a challenging task during control law implementation, due to high coupling and disturbance torque. Some states of the system during modelling are unavailable, cause parametric uncertainty for measurements. Therefore, some assumptions have been made while deriving its mathematical model. Nonlinear Dynamic Inversion (NDI) provides a simplified model of TRMS and RGDI remove some limitations of NDI as well as the singularity issue. The design and stability analysis verified through the controllability and the observability matrix. The SMC and ${H_\infty }$ strategies based on RGDI shows satisfactory convergence against considered uncertainties. The chattering phenomena reduces the performance of actuators and it may damage the actuators of the system due to fast fluctuations in control input voltage in real-time implementation. In RGDI based robust optimization control method, the weights have been selected iteratively in such a way that high gains have been achieved for the low frequency and low gain achieved for the high frequency. The weights have been selected iteratively via stability and robustness performance based analysis. Ability to reject both noise signal and external disturbance, ${H_\infty }$ optimization strategy meet the requirements of robust stability performance and validated by numerical simulations with tracking. Accurate fast-tracking and error convergence performance in worse case of perturbations (noise matrix, parametric disturbance) was the goal of this robust optimization. Some suggestions for control engineers are also evaluated from the experimental results given below.

- The experimental validation of robust control optimization shows that the TRMS behavior in real-time implementation is very sensitive depending on the exact tuning, selected weighting functions (tuning parameters).
- The controller report verifies the robust stability as well as robust performance to the modeled perturbations (uncertainty). The maximum tolerance ability against perturbation is more than 550.
- The noise signal with high amplitude causes serious contamination for the input actuators and high range frequency.

## Data Availability

All data generated or analysed during this study are included in this published article.
